# Phytohormones Trigger Drought Tolerance in Crop Plants: Outlook and Future Perspectives

**DOI:** 10.3389/fpls.2021.799318

**Published:** 2022-01-13

**Authors:** Shehzad Iqbal, Xiukang Wang, Iqra Mubeen, Muhammad Kamran, Iqra Kanwal, Gonzalo A. Díaz, Aqleem Abbas, Aasma Parveen, Muhammad Nauman Atiq, Huda Alshaya, Tarek K. Zin El-Abedin, Shah Fahad

**Affiliations:** ^1^Faculty of Agriculture Sciences, Universidad De Talca, Talca, Chile; ^2^Shaanxi Key Laboratory of Chinese Jujube, College of Life Sciences, Yan’an University, Yan’an, China; ^3^Key Lab of Integrated Crop Disease and Pest Management of Shandong Province, College of Plant Health and Medicine, Qingdao Agricultural University, Qingdao, China; ^4^School of Agriculture, Food, and Wine, The University of Adelaide, Adelaide, SA, Australia; ^5^Department of Plant Pathology, University of Agriculture, Faisalabad, Pakistan; ^6^College of Plant Science and Technology, Huazhong Agricultural University, Wuhan, China; ^7^Department of Soil Science, Faculty of Agriculture and Environmental Sciences, The Islamia University of Bahawalpur, Bahawalpur, Pakistan; ^8^Cell and Molecular Biology, University of Arkansas, Fayetteville, NC, United States; ^9^Department of Agriculture and Biosystems Engineering, Faculty of Agriculture (El-Shatby), Alexandria University, Alexandria, Egypt; ^10^Hainan Key Laboratory for Sustainable Utilization of Tropical Bioresource, College of Tropical Crops, Hainan University, Haikou, China; ^11^Department of Agronomy, The University of Haripur, Haripur, Pakistan

**Keywords:** phytohormones, drought stress, microorganisms, tolerance mechanisms, genes

## Abstract

In the past and present, human activities have been involved in triggering global warming, causing drought stresses that affect animals and plants. Plants are more defenseless against drought stress; and therefore, plant development and productive output are decreased. To decrease the effect of drought stress on plants, it is crucial to establish a plant feedback mechanism of resistance to drought. The drought reflex mechanisms include the physical stature physiology and biochemical, cellular, and molecular-based processes. Briefly, improving the root system, leaf structure, osmotic-balance, comparative water contents and stomatal adjustment are considered as most prominent features against drought resistance in crop plants. In addition, the signal transduction pathway and reactive clearance of oxygen are crucial mechanisms for coping with drought stress via calcium and phytohormones such as abscisic acid, salicylic acid, jasmonic acid, auxin, gibberellin, ethylene, brassinosteroids and peptide molecules. Furthermore, microorganisms, such as fungal and bacterial organisms, play a vital role in increasing resistance against drought stress in plants. The number of characteristic loci, transgenic methods and the application of exogenous substances [nitric oxide, (C_28_H_48_O_6_) 24-epibrassinolide, proline, and glycine betaine] are also equally important for enhancing the drought resistance of plants. In a nutshell, the current review will mainly focus on the role of phytohormones and related mechanisms involved in drought tolerance in various crop plants.

## Introduction

**FIGURE 1:**
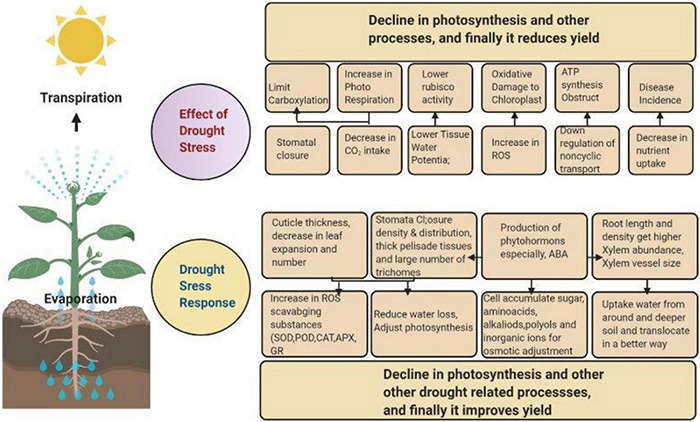
Crop response, from physiological and morphological perspectives to drought stress. Drought stress affects the normal functioning of plants by lowering the rate of photosynthesis. To overcome this issue, plants increase the production of hormones and photosynthesis-related components as a defense mechanism.

Plants are influenced by both biotic and abiotic factors, and in response to these factors, numerous internal changes occur in plants. These biotic and abiotic factors influence plant growth and development along with productivity. Biotic factors are interactions of organisms with plants that have both positive and negative effects. Positive effects may have a beneficial influence on plant growth. Negative effects may include allelopathy, herbivory influence, or pathogen infection in plants (Ciura and Kruk, 2018). Plant defense systems with various chemical compounds help to resist those negative effects (Li et al., 2019; Riaz et al., 2021), as described briefly in Figure 1. Plant cell walls are proteins (antimicrobial) and secondary metabolites reservoirs with a highly weighted molecular layer of polysaccharides that resist pathogen physical penetration and growth.

Notably, the innate immune defense system of plants limits pathogen expansion through PTI (pattern-triggered immunity), PRRs (pattern recognition receptors), and ETI (effector-triggered immunity). ETI forms lesions on plant surfaces that restrict the further movement of pathogens from the infection site (Nishiyama et al., 2013). These defense systems activate a league of defense responses against pathogens in plant infection sites (Teixeira et al., 2019). In response to biotic and abiotic factors, plants have growth regulatory hormones that play a crucial function (Ciura and Kruk, 2018; Ali et al., 2021a). Plant growth hormones, primarily known as phytohormones, are organic, natural and small lipophilic compounds. Phytohormones play a significant role in response to different biotic and abiotic stresses along with the coordination and regulation among most developmental and growth functions in plants (Jiang and Asami, 2018). Also, they regulate cellular processes and respond very effectively to external stimuli and changing environmental conditions, even at very low concentrations (Nowicka et al., 2018).

**FIGURE 2:**
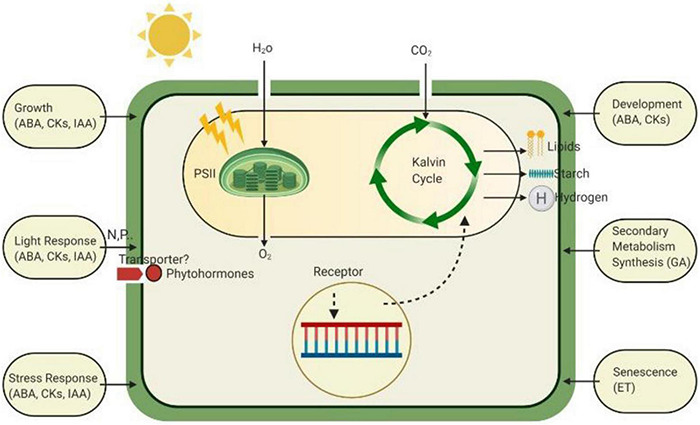
The role of various phytohormones in plants to defend against different stressed conditions by increasing or decreasing their levels. Unstable levels of these hormones work within the defense mechanism of plants to ensure healthy normal growth.

Phytohormones with low molecular weights are more frequently adopted defense mechanisms of plants to receive external stimuli precisely against biotic stresses (Cao et al., 2011; Parveen et al., 2020). Based on phytohormone physiological functions and chemical structures, there were only a few regulatory hormones, namely jasmonic acid (JA), salicylic acid (SA), ethylene (ET), auxins (IAA), gibberellins (GAs), abscisic acid (ABA), and cytokinins (CKs), which have been more often studied by plant biologists (Dubois et al., 2018). However, presently, brassinosteroids (BRs), jasmonic acid-based compounds, cytokinins based compounds (zeatin), salicylic acid-based compounds, strigolactones, and peptides are also being investigated as plant hormones (Hu et al., 2020). Based on the chemical structures of some specific groups, phytohormones are further subdivided (Péret et al., 2013) and are responsible for the formation of roots and tropism and elongation. Seed and bud dormancy occurs by inhibiting phytohormones that resist abiotic stresses, and growth becomes active after the environment becomes favorable for growth (Liu J. et al., 2019). Various derivatives of all phytohormones are present, such as transport, activated or inactivated storage forms, degradation metabolites and, most importantly, sugar or amino acid conjugates. The biological effects of many plant growth hormones are the result of the combined induction of more than one hormone. Free hormones show similar biological activity to these derivatives; therefore, a precise concentration is required for maximum effect estimations (Zhao et al., 2019), and the estimation of the effect is shown in Figure 2. Many techniques have been used for phytohormone separation to study their effects more deeply. First, thin layer chromatography techniques (Stec et al., 2016) and high-performance liquid chromatography techniques are most commonly used for plant growth hormone separation (Floková et al., 2014). GC-MS-based methods are used to profile and study phytohormone profiles in citrus species, particularly in *Citrus sinensis* L., to recognize the responses to biological and environmental stresses. Specific ionic monitoring (SIM) methods were used to evaluate the description of phytohormones. Two derivation reagents, N-Methyl-N-(trimethylsilyl) trifluoroacetamide (MSTFA) and methyl chloroformate (MCF), and one extraction solvent mixture were used. This method showed recovery with a high extraction percentage and reproducibility with a low limit of quantification and detection. With this method, they detected thirteen (13) phytohormones, such as auxins, salicylic acids, gibberellin, jasmonic acid, and abscisic acid, that belong to different groups. Jasmonic acid and auxins were only present in the vegetation of plants, abscisic acid was in the leaves and roots and salicylic acid, cytokinins and gibberellins were found in all plants. Phytohormones are present at extremely low concentrations in plants, making their analysis more difficult. Salicylic acid (SA) is the most abundant phytohormone present in various tissues, mostly ranging from 59 to 70% of growth hormones. ABA phytohormone was the highest among SA groups, and GA7 was the most abundant among all GA groups and was made from GA12 in the 3β-hydroxy gibberellic acid pathway (Farrow and Facchini, 2014).

Drought being one of the abiotic stresses, is the most compelling ecological issue that significantly damages plant photosynthesis, development and growth (Rizwan et al., 2015; Fahad et al., 2017). Perennial fruit trees and crops demand well-drained soils for healthy growth and development and to obtain the maximum level of productivity. Even for a short time, poor drainage can markedly affect the productivity of perennial fruit trees for extended durations (Fahad et al., 2017; Fuller and Stevens, 2019). Moreover, drought stress causes an imbalance in carbon metabolism, which is the primary source of carbohydrates, leading to partial stomatal closure at carboxylation sites with less carbon dioxide availability (Hu et al., 2019). In addition, drought stress also causes the shoot respiration level to increase to sustain metabolic activity. Then, a decrease in the carbohydrate reserves occurs in the storage organs of citrus plants (Fahad et al., 2017).

Moderate drought stress leads to increased leaf soluble carbohydrate concentrations, a reduction in starch concentrations and a low photosynthesis rate in leaves. Plants may use stored carbohydrates because changes in carbon availability are observed under low photosynthesis rates. To meet the plant metabolic demand, these plants stored reservoirs to overcome stressful drought conditions (Fahad et al., 2017; Liu Y. N. et al., 2019). However, severe drought stress reduces starch and soluble fraction levels (Rizwan et al., 2015; Fahad et al., 2017). Plants exposed to saline environments experience a decline in plant growth because of the effect of specific ions on metabolism and antagonistic environmental connections. Various technologies have been used to exploit citrus plant growth under drought stress. Attempts are being made through conventional breeding methods to improve plants tolerance to drought stress, and these methods are laborious and based on the prevailing genetic changeability. Recently various drought resistance genes were overexpressed in plants, and plants become tolerant to drought stress. In most cases, a higher yield was recorded in the transgenic plants than the wild type plants. Some of the drought-tolerant plants have been listed in Table 1.

**TABLE 1:** Transgenic crops under drought stress experienced altered yields through the phytohormone signaling pathway.

Gene(s)	Expressing plant	Signaling pathway	Stress type	Environmental condition(s)	Effect on yield (increase (%)	References
*AtYUCCA6*	*Solanum tuberosum*	Auxin	Drought	Greenhouse	Data not shown	Im Kim et al., 2013
*LOS5ABA3*	*Glycine max*	ABA	Drought	Growth chamber and field	21%	Li et al., 2013
*AtEDT1/HDG11*	*Oryza sativa*	ABA	Drought	Greenhouse and field	16%	Yu et al., 2013
*AtGAMT1*	*Solanum lycopersicum*	GAs	Drought	Greenhouse	Data not shown	Nir et al., 2014
*AtEDT1/HDG11*	*Gossipium hirsutum*	ABA	Drought and salinity	Laboratory, greenhouse and field	43%	Yu et al., 2016
*GhABF2*	*Gossipium hirsutum*	ABA	Drought and salinity	Laboratory, greenhouse, and field	46%	Liang et al., 2016
*JIOsPR10*	*Oryza sativa*	JA	Drought, salt and *Magnaporthe oryzae*	Greenhouse	No change	Wu et al., 2016
*GA2ox*	*Oryza sativa*	GAs	Drought and disease	Greenhouse and field	10–30%	Lo et al., 2017
*OsERF109*	*Oryza sativa*	JA and ABA	Drought	Greenhouse	30–45%	Yu et al., 2016

In response to abiotic stresses, i.e., drought and salinity, plants have developed various physiology, phenology, morphology, and biochemical-based mechanisms to sustain their cellular osmotic potential Fahad et al., 2015, 2017, 2018, 2020, 2021a,b,c,d,e,f; Kamran et al., 2019; Ali et al., 2021a). Various studies on these mechanisms are ongoing due to the involvement of multiple phytohormones acting as sole mediators for avoidance, tolerance, and the adverse effect of water stress. Plant hormones vitally regulate the development and growth of plants along with drought stress reflexes throughout the lifespans of plants (Sah et al., 2016; Ullah et al., 2017). In response to drought stress, plants produce phytohormones that transduce the pathway to regulate its impact (Fahad et al., 2017; Hamayun et al., 2018). Also, phytohormones activate different developmental and physiological processes, such as negative phototropism in roots, osmotic balance, and closing stomata (Lim et al., 2015; Zahid et al., 2016).

Exogenous applications of plant growth regulators are also employed to overcome these stressed conditions (Kamran et al., 2021). As mentioned, phytohormones were applied to improve drought tolerance in plants (see Table 2) and increase growth, development, and productivity. Phytohormones play important roles in modifying the plant reflex to strains at very low concentrations, and their chemical messenger properties are produced in one part of the plant and transferred to entire parts of plants. Phytohormones are natural products synthesized chemically as plant growth regulators (Sah et al., 2016).

**TABLE 2:** Phytohormones functions to prevent plants from drought stress.

Functions	Hormones	References
Involved in cell division, cell elongation, apical dominance, phyllotaxis and tropic responses	Auxin	Im Kim et al., 2013
Root branching	Auxin	Im Kim et al., 2013
Growth of plant parts and the flowering stage	Cytokinins	Liang et al., 2016
Development of female gametes and embryos	Cytokinins	Wu et al., 2016
Photomorphogenesis and leaf senescence	Cytokinins	Ha et al., 2012
Cell elongation and increasing the cell division	Gibberellins	Colebrook et al., 2014a
Enhance the vegetative and reproductive stages of plants	Gibberellins	Li et al., 2012a
Stomatal closure, gene upregulation and compatible osmolyte synthesis	Abscisic acid (ABA)	Shi et al., 2018
Photosynthetic activity, stomatal regulation, root growth, and germination	Abscisic acid (ABA)	Seo and Koshiba, 2011
Defense responses	Salicylic acid (SA)	Miura et al., 2013
Progressive responses against elevated temperature stress	Salicylic acid (SA)	Munne-Bosch and Penuelas, 2003
Stomatal closure	Salicylic acid (SA)	Dempsey et al., 2011a
Drought tolerance by lessening transpiration, squeezing the aperture of the stomata, and thinning the cuticle	Ethylene	Zandalinas et al., 2016
Appraisal of growth, drought tolerance, and yields	Brassinosteroids	Li et al., 2012b
Stomatal closing	Jasmonic Acid (JA)	De Ollas et al., 2013
Increase the antioxidant activity of plants under drought	Jasmonic Acid (JA)	Dong and Hwang, 2014
Root growth, pollen tube growth, stomatal development	Peptides	Shi et al., 2018

## Plant Hormones Improve Drought Resistance in Plants

### Auxins

Auxin is an important phytohormone. Auxins are involved in cell division, cell elongation and the differentiation of cellular tissues, embryogenesis, root formation, apical dominance, phyllotaxis, and tropic responses. Auxin genes are important biotechnological targets for modifying plant size and shape and improving plant yield. Therefore, they play a vital role in cell and growth development (Asgher et al., 2015). Auxins also play a dynamic role in mediating and improving plant tolerance to non-infectious stresses, such as deficiency conditions, as represented by many research reports (Kazan, 2013). Indole-3-acetic acid was one of the first hormones recognized in this group and is most commonly found among the auxins (Hamayun et al., 2021c). Indole 3-acetic acid is produced from tryptophan and is chemically similar to it. Alterations in gene expression patterns were used to control auxin-mediated growth and development. When plants are exposed to drought and other stress conditions, varied modulations in the synthesis, metabolism, transport, and activity of auxins take place, as depicted in various reports (Ljung, 2013). A decline in the IAA level under stress conditions can increase the ABA level in plants to induce growth modulation by auxins. Jung et al. (2015) mentioned that among auxin-coding genes recognized in rice plants, some genes were activated by drought stress. Previous studies also reported the overexpression of YUC6 in poplar and potato, resulting in auxin-enhanced drought tolerance and phenotypes (Ke et al., 2015). Auxins also promote root branching and have a potential role in drought tolerance mechanisms in tobacco seedlings (Verma et al., 2016; Wang et al., 2018). Auxin response factors (ARFs) bind directly to the promoters of auxin-responsive genes, allowing them to be activated or repressed transcriptionally and enhance stress tolerance in tomatoes (Bouzroud et al., 2018). In addition, these ARFs regulate genes *(WRKY108715*, *MYB14*, *DREB4*, and *bZIP 107*) involved in drought stress response and enhanced tolerance in clovers (Zhang et al., 2020).

The role of auxin in drought stress has been explored via TLD1/OsGH3.13 which encodes indole-3-acetic acid (IAA)-amido synthase; it then enhanced the expression of late embryogenesis abundant (LEA) genes, which then increase the resistance in plants against drought stress. In addition, genes Aux/IAA genes were identified in rice and most of these genes were expressed under drought stress. In a study, YUC6 was overexpressed in potato and poplar which showed auxin-overproduction phenotypes and enhanced drought tolerance (Colebrook et al., 2014b). Auxin also enhanced drought resistance by interacting with other phytohormones. For example, auxin regulates various members of the ACS (1-aminocyclopropane-1-carboxylate synthase) gene family, which is a rate-limiting enzyme in ethylene biosynthesis. This interaction enhances resistance in plants against drought stress (Colebrook et al., 2014b).

### Cytokinin

Cytokinins were discovered in 1950, and they are the most important phytohormones that stimulate cell division and induce variations. The first natural cytokinin was *trans*-zeatin, which was isolated from maize (Miller, 1961). These compounds are adenine byproducts derived from isoprene or an aromatic side chain at the *N6* position of purine. Folke Skoog and his assistants isolated kinetin (the cytokinesis-promoting factor) from autoclaved herring sperm DNA (Miller, 1961). These hormones are essential for the growth of plant regulation and acclimation to drought stress (Li et al., 2016). Cytokinins have both negative and positive impacts on drought stress (Ha et al., 2012; Li et al., 2016).

The enhancement or reduction of the cytokinin level depends on the period and severity of the drought stress (Zwack and Rashotte, 2015). The beneficial aspects are enhanced intolerance against drought stress. CKs are also reported to stimulate transgene expression in transgenic plants, i.e., isopentenyl transferase gene expression. The transgenic plants indicated significant drought tolerance through delayed senescence by restricting drought-induced leaf senescence. The negative effects of CK accumulation on drought tolerance have also been reported along with the positive effects of CK accumulation. CK oxidase/dehydrogenase (CKX) catalyzes CK and is involved in the overexpression and breakdown of CKX in *Arabidopsis*, which results in a decrease in endogenous CK contents (Werner et al., 2010; Hamayun et al., 2021b). Therefore, *CKX1, CKX2, CKX3, and CKX4* were overexpressed independently in *Arabidopsis*, resulting in transgenic lines with reduced CK levels and subsequently greater drought tolerance.

Cytokinins are helpful in plant tissue culture techniques and support the thoughtful study of plant biological processes, such as the growth of plant parts and the flowering stage. These compounds are responsible for stimulating different processes during the growth and development of female gametes and embryos of a plant. Notably, cytokinins also participate in seed germination, vascular development, photomorphogenesis, shoot apical meristem development, floral development, and leaf senescence. It also helps plants to induce adaptive responses to drought and adverse ecological conditions (Mao et al., 2020). Moreover, different hormones and macronutrients control the transcription of cytokinin biosynthetic genes. In *Arabidopsis*, cytokinins stimulate cell division by antagonizing auxin. Auxin promotes the expression of *AtIPT5* and *AtIPT7*, whereas cytokinins suppress the expression of *AtIPT1*, *AtIPT3*, *AtIPT5*, and *AtIPT7* in the shoot meristem (Ismail H. M. et al., 2020).

All the genes related to cytokinin in *Arabidopsis* were overexpressed individually, and transgenic lines of *Arabidopsis* with decreased levels of cytokinin gradually improved tolerance to drought conditions (Nishiyama et al., 2011). The current need is to elucidate the signaling and role of cytokinins under drought conditions.

### Gibberellins

Gibberellins are tetracyclic diterpenoids of carboxylic acids. The primary purpose of GAs in plants is as growth hormones and to provide resistance against drought stress and other abiotic stresses. GAs continues their functions in plants throughout the plant life cycle. The primary purpose of gibberellins is to enhance the development of plant tissues by cell elongation and increasing the cell division process enhances the immature and adult stages of plant growth. It also helps to enhance the vegetative and reproductive stages of plants (Colebrook et al., 2014b; Kang et al., 2019).

*SIDREB* (drought-responsive element-binding protein) increases drought tolerance in tomatoes by decreasing the expression of gibberellin biosynthesis genes (Colebrook et al., 2014a). Drought tolerance is said to be enhanced if the GA level is reduced in plants. Transgenic tomatoes are produced by the overexpression of the *AtGAMT1* (*Arabidopsis thaliana* GA Methyl Transferase-1) gene. *AtGAMT1* encodes an enzyme that causes a breakdown in the methylation of active GA to make inactive GA methyl esters. The transgenic tomato indicated a reduction in gibberellins by enhanced drought tolerance. An increased water level in leaves was observed in transgenic tomatoes under drought stress because of transpiration in plants (Nir et al., 2014). The ectopic expression of GA2ox (GA 2-oxidase) increased drought tolerance. This protein also helps to enhance resistance in rice plants (Lo et al., 2017). The *DELLAs* proteins are the primary stimulators of GA responses in drought conditions faced by plants. The functions of this group of nuclear regulators are to suppress gibberellin stimulation in plants. Gibberellins binding to the receptor GID1 (GA in-sensitive dwarf 1) lead to the degradation of *DELLAs* by the 26S proteasome and the stimulation of gibberellin responses (Li et al., 2012c).

Drought tolerance in plants is appreciably enhanced by gibberellins, as reported in many studies (Li et al., 2012a; Colebrook et al., 2014a). Tomatoes make the transgene by overexpressing the gene (methyltransferase 1). *GAMT1* encodes an enzyme catalyzing active gibberellin methylation to form inactivated GA methyltransferase in *Arabidopsis* spp. The resulting tomato plant expressed a typical GA-deficient phenotype, which showed drought tolerance. A high-water content was prominent in transgene tomato plants because of decreased transpiration (Ullah et al., 2018). By contrast, applying hormones resulted in the reappearance of normal growth, and plants became prone to drought again (Nir et al., 2014). In addition, the external expression of GA oxidase enzyme (GA 2-oxidase) improved drought and disease hindrance in rice plants (Lo et al., 2017). *SIDREB* (drought-reflexing binding protein) also improved drought tolerance in tomatoes by suppressing the genes involved in GA biosynthesis (Li et al., 2012a). DELLAs protein factors also primarily regulate response to GA, and this group of nuclear regulators especially act to suppress the GA response. GA binding to the insensitive dwarf1 receptor of GA will result in *DELLA* degradation, similar to 26S proteasome and GA response stimulation (Li et al., 2013).

### Abscisic Acid

Abscisic acid is an important signaling phytohormone under drought stress (De Ollas et al., 2013). Abscisic acid plays a significant role in regulating stomatal closure, gene upregulation and compatible osmolyte synthesis. 9-Neoxanthin cis-epoxicarotenoid dioxygenase (NCED) is used in abscisic acid synthesis and is considered a bottleneck, covering 9-neoxanthins to xanthins. This enzyme is used in the upregulation of an increased level of abscisic acid. Abscisic acid is cleaved into 8′-OH-ABA, and this process is catalyzed by an ABA 8′-hydroxylase (CYP707A) enzyme. This reaction is used to inactivate abscisic acid. ABA 8′-hydroxylase compounds are spontaneously converted into dehydrophaseic acid (DPA) and phaseic acid (PA), the primary degradation products. Another path that is used to inactivate abscisic acid pools is through conjugation to hexoses catalyzed by the ABA O-glycosyl transferase enzyme. This process yielded the ABA-glycosyl ester (ABAGE) compound (Dong and Hwang, 2014). After the cleavage of the ABA glycosyl ester (ABAGE) compound by an ABA glycosyl ester β-glycosidase (BG18) enzyme, active abscisic acid is released. Both species have increased levels of phasic acid (PA) and dehydrophaseic acid (DPA) under drought stress, but Cleopatra exhibited a more increased level of ABA-glycosyl ester (ABAGE).

**FIGURE 3:**
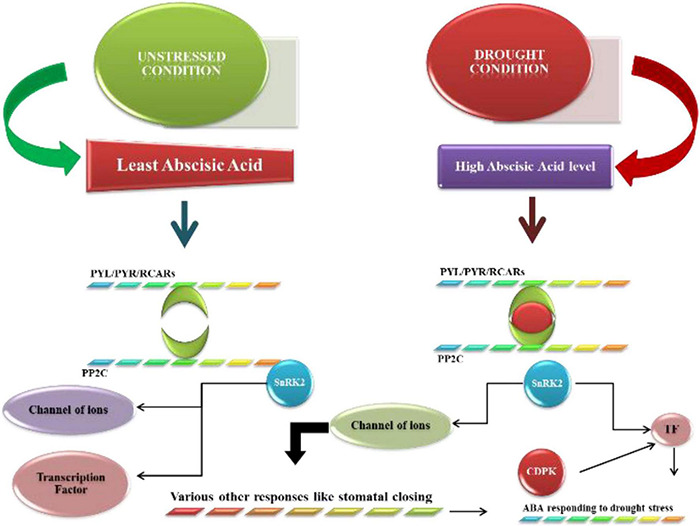
ABA’s fundamental signaling mechanism during stressful situations. The ABA levels are lower under ideal circumstances, and the function of SnRK2 protein kinase is blocked by PP2C phosphatases. The cellular ABA level rises during high-pressure situations, and ABA then attaches to PYR/PYL/RCARs, which connect to and deactivate PP2Cs in response. If they are detached from PP2C, then SnRK2s are automatically activated. Switched on SnRK2s phosphorylate the following targets and provoke molecular and physiological reactions through ABA.

Abscisic acid signaling pathways have vital role in the expression of drought stress-responsive genes because various stress situations can occur in plants. Abscisic acid receptors are very important in the transduction of signals. In the subcellular state, many receptors are recognized. Under normal conditions, ABA is expressed at low concentrations in plants (Parveen et al., 2021). SnRK2 activity in protein kinases is inhibited by the phosphatase PP_2_C, leading to dephosphorylation. As plants develop ABA concentrations, they start to bind with PYRs, PYLs, and RCARs, which bind to deactivate PP_2_C phosphatase activity (Danquah et al., 2014). These PYRs and PYLs are essential receptors for the abscisic acid response encoded by different genes, such as 11 genes in rice and 14 genes in *Arabidopsis* encoding these receptors (Klingler et al., 2010). The autoactivation of SnRK_2_ (protein kinases that enhance the abscisic acid response) occurs when the protein is dissociated from phosphatase PP_2_Cs (Hrabak et al., 2003; Ullah et al., 2017), and the rest of the SnRKs are involved in the abscisic acid response (SnRK2.2, SnRK2.3, and SnRK2.6) (Feng et al., 2014). In *Arabidopsis*, only the A clade participates in the signaling of abscisic acid out of seventy-six PP_2_Cs. Additionally, the ABA signaling pathway is also dependent on branches of various transcription factors, such as MYC, NAC and MYBs. The responsive elements of abscisic acid also play a role in ABA signaling (De Ollas and Dodd, 2016). CDPKs (CDPKs) also proved very important in the pathway related to signaling. Calcium-dependent protein kinases also participate in ABA signaling, and 34 CDPKs have been reported in *Arabidopsis*, along with 29 in rice, 20 in wheat and 35 in maize. Two CDPKs, CPK4, and CPK11, have been reported to be involved in the regulation of ABA signaling in *Arabidopsis*. SnRK_2_ activation triggers the phosphorylation of down–target genes, resulting in the induction of molecular and physiological responses to ABA triggering, such as photosynthetic activity, stomatal regulation, root growth, and germination (Altaf et al., 2020). Abscisic acid also regulates many other genes related to drought stress to develop drought tolerance in plants. The abscisic acid-induced pathway is shown in Figure 3.

### Salicylic Acid

**FIGURE 4:**
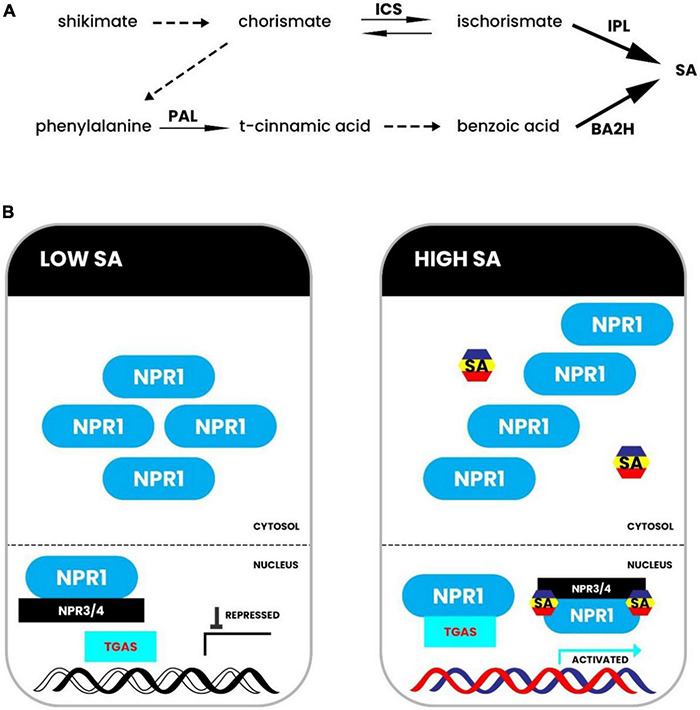
Biosynthesis and signaling of salicylic acid. **(A)** In *Arabidopsis*, a model for salicylic acid (SA) production has been proposed. Genetic investigations showed the isochorismate pathway in the upper panel. Biochemical research showed the phenylpropanoid pathway in the lower panel. **(B)** NPR1 forms an oligomer in the cytosol in cells with low SA levels, and NPR3 and NPR4 bind leftover NPR1 in the nucleus to block NPR1 activity. NPR1 becomes monomeric and reaches the nucleus in cells with high SA levels, where SA binds to NPR3 and NPR4 to disrupt their transcriptional repression action. NPR1 interacts with TGAs in SA-responsive promoters, causing defensive responses to be activated. BA2H stands for benzoic acid 2-hydroxylase; ICS stands for isochorismate synthase; IPL stands for isochorismate pyruvate lyase; NPR stands for non-expresser of pathogenesis-related genes; PAL stands for phenylalanine ammonia lyase; SA stands for salicylic acid; TGA stands for TGACG-binding factor. Adapted from Li et al. (2019).

The salicylic acid (SA) hormone is associated with drought tolerance and signaling in plants (Miura and Tada, 2014). In response to drought stress, SA biosynthesis takes place via both the isochorismate pathway and the phenylpropanoid pathway. Both pathways require the chemical chorismate generated from the shikimate pathway (Figure 4A). However, the isochorismate pathway is known to be the major pathway in most plants (De Ollas et al., 2013). The phenylpropanoid pathway for SA biosynthesis begins with phenylalanine (Phe) being converted to trans-cinnamic acid (t-CA) by phenylalanine ammonia lyase (PAL). It is then transformed into benzoic acid (BA). Researchers have yet to discover the enzyme that converts t-CA to benzoic acid. After that, the enzyme benzoic acid 2-hydroxylase catalyzes the hydroxylation reaction that yields SA from BA (BA2H) (Figure 4A). The isochorismate pathway requires enzyme ICS1 (isochorismate synthase 1) that converts chorismate to isochorismate, and isochorismate is then converted to SA by isochorismate pyruvate lyase (IPL) (Danquah et al., 2014). Salicylic acid also plays a significant role in defense responses against drought stress, as shown in Figure 4B. NPR1 (non-expresser of pathogenesis-related genes 1) is a master regulator of defensive signals mediated by SA. SA binds to NPR1 and NPR1 homologs directly, perhaps regulating NPR1 activity and stability. Increased cellular SA levels cause a redox shift in the cytoplasm, causing NPR1 to transition from oligomer to monomer forms (Figure 4B). The active monomers then go to the nucleus, where SA binds to NPR3 and NPR4 to block their transcriptional repression activity. NPR1 interacts with TGAs (TGACG-binding factors), activating defense responses against drought stress. However, in cells with low SA levels, NPR1 forms oligomers and persists in the cytosol, while NPR3 and NPR4 bind residual NPR1 in the nucleus to block NPR1 function (Figure 4B; Dempsey et al., 2011b). Salicylic acid accumulation in plants improves responses to various abiotic stress conditions, such as drought and salinity (Miura and Tada, 2014), antioxidant activity and photosynthetic machinery protection, which prevents electron leakage (Ismail I. et al., 2020; Malik et al., 2021). Previous studies showed that drought increased endogenous levels of abscisic acid (ABA) and jasmonic acid (JA) in *Brassica napus*, resulting in a rise in ABA/SA and (ABA + JA)/SA (Lee et al., 2019). The increase of ROS and proline, as well as a loss of reducing potential [NAD(P)H/NAD(P) + and GSH/GSSG], paralleled the changes in endogenous hormonal balance. Drought-induced O_2_ buildup was scavenged by SA pretreatment (Lee et al., 2019). Moreover, drought increased ROS generation and, as a result, lipid peroxidation, which is a specific indicator of oxidative damage. On the other hand, Exogenous SA application substantially reduced oxidative damage in rice seedlings in hydroponic and soil systems by upregulating antioxidant enzymes (Ali et al., 2021b). Furthermore, drought stress lowered photosynthetic pigment concentration, gas exchange parameters, proline, soluble sugars, total phenolic, flavonoids, growth, and biomass output. On the other hand, SA promoted *Portulaca oleracea* growth and biomass production by improving photosynthetic pigments, gas exchanges, suitable solutes, and secondary metabolites (Hamayun et al., 2021a). In *Impatiens walleriana*, dehydration increased the quantity of electrolyte leakage (EL), malondialdehyde (MDA), peroxidase (POD), and ascorbate peroxidase (APX) activities, along with proline content. P5CR (gene for 1-pyrroline-5-carboxylate reductase) has a similar expression pattern to P5CS, with minor changes in intensity. Through improved antioxidant activity and water balance, SA lowered the amount of EL and MDA in the plant (Safari et al., 2021). When compared to non-SA pretreated *Brassica rapa*, SA pretreatment dramatically boosted proline concentration via upregulating the expression of genes expressing pyrroline-5-carboxylate synthase (P5CSA and P5CSB) and down-regulating the expression of the gene encoding proline dehydrogenase (PDH). In another case, the Carrizo citrus plant variety showed greater tolerance to drought and heat stress in combination. Drought stress increased salicylic acid levels in the leaves of certain citrus species, such as Carrizo and Cleopatra. Chlorophyll fluorescence, gas exchange parameters and malondialdehyde (MDA) accumulation indicate that Cleopatra mandarin is susceptible to drought and heat stress (Hamayun et al., 2018). Phenotypic traits of citrus plants occur in response to a combination of drought and heat stress. Whole sprouts (%) of Cleopatra and Carrizo seedlings were exposed to drought and heat stress (40°C) in combination for 10 days. For each genotype, asterisks denote statistical significance concerning the initial values at *P* ≤ 0.05 (Hamayun et al., 2021a).

The salicylic acid levels also increased in plants under drought stress, which may be five times that of the normal level recorded in evergreen shrubby plants in *Phillyrea augustifolia* (Munné-Bosch and Peñuelas, 2003; Hamayun et al., 2021c). The enhanced drought tolerance and disorder resistance found in mutants of *Arabidopsis* spp. such as adr_1_, acd_6_, myb_96–1_, and cpr_5_ are due to the presence of salicylic acid (Seo and Koshiba, 2011; Miura et al., 2013). In *Arabidopsis*, stomatal closing was also observed due to salicylic acid accumulation under stressed conditions because the SA-regulated induction of *PR* gene expression led to drought tolerance by shutting the stomatal openings (Liu et al., 2013; Miura et al., 2013), and stomatal closure occurred through the accumulation of SA under the influence of SIZI in *Arabidopsis*, significantly increasing the drought tolerance.

In another study, the exogenous application of SA positively regulated *ICS1* gene (isochorismate synthase) and enhanced drought tolerance in *Arabidopsis*. Furthermore, it revealed that SA activated *WRKYs* and *TGAs* genes, which then enhanced the plant immune system against drought stress (Klingler et al., 2010).

### Jasmonic Acid

**FIGURE 5:**
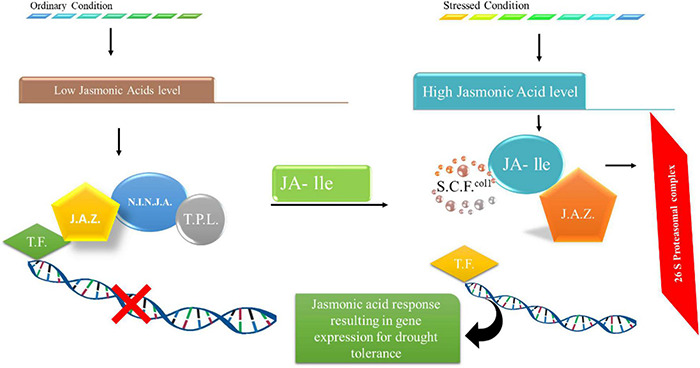
Changes in JA signals under stressful situations. JAI3/JAZ proteins attach to different transcription components and restrict their function along with reduced levels of JA under ideal circumstances. The JA concentrations are high and deteriorate JAZ proteins under stressful situations, leading to the activation of transcriptional components that upregulate genes associated with stress responses.

Drought tolerance in plants is induced by closing stomata, shifting reactive oxygen species, and deep root growth in the case of jasmonic acid. Studies have revealed that JAs participate in stomatal closing regulation as a result of drought stress (Riemann et al., 2015). A case study revealed that treating *Arabidopsis* with 12-OPDA led to stomatal shutting. It is also involved in indirectly decreasing stomatal gaps, favoring drought tolerance. Drought also prevents the alteration of OPDAs to jasmonic acid; in this case, OPDA coupled with ABA or individually leads to the closing of stomata (Savchenko et al., 2014; Kazan, 2015). High ROS foraging was found (Fang et al., 2016) in transgenic plants overexpressing VaNAC26, which showed relatively more drought tolerance. JA-related genes were highly regulated in overexpressed lines under drought and ordinary conditions. The external application of JA led to a perfect reflex by plants to drought stress. External JA application was also shown to enhance the activity of antioxidants under drought stress (Shan et al., 2015). Another case revealed that JA was found to be an enhancer of different enzymes in young wheat, such as ascorbate peroxidases (APX) and ascorbate reductases monodehydroascorbate reductase and glutathione reductase, under stress conditions (Shan et al., 2015). JA also plays a significant role in water conductivity from soil under restricted moisture conditions (De Ollas et al., 2013; Sánchez-Romera et al., 2014) found that the transient presence of jasmonic acid in roots is required under drought stress to increase the abscisic acid levels. However, the function of JA still must be clarified under drought stress to obtain highly tolerant plants. The exogenous application of JA increased the antioxidant activity of plants under drought conditions, as shown in Figure 5. The proteins JAI3/JAZ bind to various transcription factors, including MYC2, and limit their activity under normal conditions. However, during drought stress, the degradation of JAZ proteins occurs, resulting in active transcription factors that upregulate genes of JA, which enhances tolerance in plants against drought stress (De Ollas and Dodd, 2016). Moreover, JA signaling pathways interact with ABA signaling pathways, suggesting their role in response to drought stress. It has been recently revealed that JA enhanced the hydraulic conductivity of plant roots under drought stress by interacting with calcium and ABA-dependent and independent signaling pathways (De Ollas and Dodd, 2016).

### Brassinosteroids

Brassinosteroids play a role in stress responses along with roles in plant growth and nourishment. These stresses include drought, cold, hypersalinity, metallic heaviness, raised temperature, and infectious agents (Chen et al., 2017). It was previously mentioned that in *Arabidopsis*, wheat, and *Brassica* spp., brassinosteroids played a positive role in response to drought stress (Divi et al., 2010). *Arabidopsis* biosynthetic gene (DWF_4_) overexpression resulted in the appraisal of growth, drought tolerance, and yields (Sahni et al., 2016). By contrast, there was a negative response from the brassinosteroids. Additionally, mutants of Brs showed functional loss, but increased drought tolerance (Northey et al., 2016; Ye et al., 2017), and knocking out BR_1_ by miRNA technique led to more drought tolerance in *Brachypodium distachyon* (Feng et al., 2015). These phytohormones play a crucial role in drought stress tolerance. They act synergistically in response to drought stress.

Recently, various WRKY transcription factors have been discovered in *Arabidopsis thaliana* and these transcription factors are reported to be involved in plant growth and response to drought stress. To regulate plant growth, BRs extensively interact with these transcription factors and GA in response to drought stress (Clarke et al., 2004; Xue et al., 2013; Sánchez-Romera et al., 2014; Sánchez-Martín et al., 2015).

### Ethylene

Ethylene was found to be actively involved in enhancing drought tolerance in plants. In a study on cotton, ethylene-responsive genes or binding protein elements such as AP_2_, EREBPs, and APETELA_2_ were identified in response to heat and drought stress (Liu and Zhang, 2017). One analysis revealed that members of ERFs (responsive factors of ethylene), GmERF_3_ isolated from *Glycine max*, whose expression was induced by drought stress, ABA, SA, JA, and ET. GmERF_3_ in overexpressing tobacco plants exhibited more tolerance to drought stress because of the high contents of proline and solubilized sugar compared to wild plants. Transgenic plants showed increased resistance to *Ralstonia solancearum, Alternaria alternata*, and tobacco mosaic virus due to the high expression of pathogenesis-related protein-coding genes (Hamayun et al., 2019). The overexpression of the ERF gene (*ERF019*) in *Arabidopsis* delayed aging and flowering time. Transgenes also exhibit drought tolerance by lessening transpiration, squeezing the aperture of the stomata, and thinning the cuticle (Scarpeci et al., 2017). In signal transduction, *ETR*_1_ codes for receptors of ET but negatively regulates the response; other receptors of this family always close down the signaling, whether ethylene is present or not (Shi et al., 2016) of the other members of the family include *ERS_1_, ETR_2_, EIN_4_*, and *ERS_2_. CTR_1_* regulates ET signaling. In *Arabidopsis*, *CTR*_1_ is involved in ET signaling, while in tomato 3, *CTR*_1_ is involved in ET signaling. There is no evidence of a decrease in the stream substrate of *CTR*_1_. When the receptor finds ethylene, it shuts off the activity of ethylene, thus leading to a reflex action for tolerance to drought conditions.

### Peptides

Recently several secreted peptides were found to mediate the cellular development in plants. However, it was unclear whether these peptides mediate long-distance signaling in response to drought stress. Among the peptides, CLAVATA3 (CLV3) is a well-characterized plant peptide involved in shoot apical meristem formation. In land plants, phytohormone abscisic acid plays a significant role in the regulation of stomatal movement to prevent water loss. However, no mobile signaling molecules have yet been discovered that can enhance the abscisic acid accumulation in leaves (Nir et al., 2014). Recently, CLE25 peptide was found to transmit water-deficiency signals through vascular tissues in Arabidopsis and affects abscisic acid biosynthesis and stomatal control of transpiration. The gene related to these peptides was expressed in the vascular tissues and enhanced root response to drought stress. These peptides move from the roots to the leaves and induce stomatal closure by modulating abscisic acid accumulation, enhancing resistance to drought stress. Recently, another peptide gene in rice, OsDSSR1, was discovered, which was expressed mainly in the root, stem, node, leaf, and panicle, and this expression was induced by drought stress. The peptide is localized in the nucleus and cytoplasm and exhibited enhanced drought stress tolerance and decreased ABA sensitivity compared to the wild type (Yu et al., 2016). Other peptides such as phytosulfokine (PSK), a growth related to cell proliferation; rapid alkalinization factor (RALF), which regulates root growth; LUREs, which guides pollen tube growth; STOMAGEN, which is related to stomatal development; and casparian strip integrity factor (CIF), which is associated with the formation of the casparian strip diffusion barrier; Another peptide, AtPep3 which plays an important role in the drought and salinity stresses were also recently discovered (Nir et al., 2014; Liang et al., 2016; Yu et al., 2016).

## Conclusion and Future Perspectives

An ever-growing population and diminishing natural resources have made it difficult for farmers to produce enough food to meet their needs. Drought is a major constraint on crop productivity worldwide and is expected to worsen in the near future. Besides, droughts are becoming more common, severe, and widespread due to climate change. Hence, scientists are attempting to develop drought-tolerant crops and understand different drought tolerance mechanisms. This review’s underlying theme is that plants typically respond to drought stress by adjusting the levels of phytohormones, such as abscisic acid, jasmonic acid, auxin, ethylene, and gibberellin, cytokinin, brassinosteroids, and small peptide molecules. These phytohormones trigger tolerance to drought stress via regulation of various morphological, physiological, biochemical and molecular processes. The morphological and physiological processes involve changes in the composition of the leaves, root growth and stomatal control. The biochemical process includes adjusting the levels of phytohormones. Molecular processes include phytohormone-mediated signals, leading to the activation of various transcription factors that causes the expression of genes required for plant survival in drought stress (Saleem et al., 2020).

However, due to the high level of complexity, most of the mechanisms by which phytohormones trigger drought tolerance in crops are poorly understood and requires further study. Besides, scientists could not comprehend the crosstalk among the phytohormones against drought stress. Since crosstalk is so intricate, the underlying mechanisms are also unknown and need further investigation. Recently scientists are attempting to understand the mechanisms of drought tolerance in plants through the exogenous application of phytohormones. In addition, drought stress in plants is alleviated by applying plant microbiomes. These plant microbiomes induce drought stress-responsive genes and play a crucial role in the acquisition of drought tolerance (Ismail A. H. et al., 2020). In the future, newly developed large-scale OMIC methods and high-throughput bioinformatic analysis will be used to seek a better understanding of the mechanisms by which phytohormones trigger drought tolerance in crops, which will ultimately lead to the development of drought-resistant crop plants with significant agronomic features.

## Author Contributions

SI, XW, IM, and MK wrote the manuscript. SI, MK, and XW designed the study idea and applied the mode. GD, AP, SF, HA, TZ, and AA collected and arranged the whole database. AA, IK, MK, and MNA analyzed and discussed the contents of the manuscript. SF, MK, IK, and SI critically revised the manuscript. All authors contributed to the article and approved the submitted version.

## Conflict of Interest

The authors declare that the research was conducted in the absence of any commercial or financial relationships that could be construed as a potential conflict of interest.

## Publisher’s Note

All claims expressed in this article are solely those of the authors and do not necessarily represent those of their affiliated organizations, or those of the publisher, the editors and the reviewers. Any product that may be evaluated in this article, or claim that may be made by its manufacturer, is not guaranteed or endorsed by the publisher.
